# Heparin-like effect contributes to the coagulopathy in patients with acute liver failure undergoing liver transplantation

**DOI:** 10.1111/j.1478-3231.2009.01977.x

**Published:** 2009-05

**Authors:** Marco Senzolo, Seema Agarwal, Paola Zappoli, Sushang Vibhakorn, Susan Mallett, Andrew K Burroughs

**Affiliations:** 1 Department of Surgical and Gastroenterological Sciences, University Hospital of PaduaPadua, Italy; 2 Royal Free HospitalLondon UK; 3 Department of Surgery, The Royal Free Sheila Sherlock Liver CentreLondon, UK; 4 Department of Anesthesia, Royal Free HospitalLondon, UK

**Keywords:** acute liver failure, coagulation, heparin-like effect, liver transplantation, thromboelastography

## Abstract

**Introduction:**

Liver transplantation (LT) in cirrhotics is characterized by severe coagulopathy, associated with a well documented heparin-like effect (HLE) seen by thromboelastography (TEG™) after reperfusion. The amount of HLE present in patients with acute liver failure (ALF) and its role in their bleeding tendency before LT has not been investigated.

**Aim:**

To investigate the presence and extent of HLE in patients with ALF undergoing LT and to compare the extent of HLE in this group with a group of cirrhotics undergoing LT.

**Material and Methods:**

Ten consecutive ALF and 10 cirrhotic patients undergoing LT were included. TEG™ (with and without heparinase I), surrogate total thrombin generation (TTG) derived by TEG™ and haemodynamic variables were recorded for every stage of the LT. HLE was defined as a correction of *r*+*k* times on TEG™ of more than 50% by the addition of heparinase I.

**Results:**

Before incision, patients with ALF showed a significantly greater HLE compared with patients with cirrhosis (*r*+*k* time: 66 min corrected to 29 vs 45 min corrected to 32 min, *P*=0.001). After reperfusion, all the patients showed extensive HLE, without any difference between the two groups. Despite the greater HLE, patients with ALF showed similar TTG compared with the cirrhotic group. By the end of the operation, the extent of the HLE was greatly reduced in both the groups.

**Conclusions:**

Before transplantation, patients with ALF have a greater HLE than patients with liver cirrhosis. However, this did not affect the thrombin generation calculated by TEG™ and resolved after transplantation.

Acute liver failure (ALF) is characterized by severe and sudden hepatic dysfunction in a previously healthy individual, resulting in a high mortality despite the use of liver transplantation (LT). Complications are hepatic encephalopathy, jaundice and severe coagulopathy. Multiorgan failure is common (1). The coagulopathy comprises a wide spectrum of haemostatic changes, including thrombocytopaenia, reduced platelet function and reduced concentrations of many coagulation factors (2). Concentrations of coagulation factors and other changes in coagulation are used prognostically as indications for LT: King's college criteria includes prothrombin time (PT) (3) and the Clichy criteria uses factor V concentrations (4). Although a prolongation of PT is due to the poor synthetic function of the liver and results in ‘anticoagulation’, patients with ALF can show microvascular thrombi within the hepatic vessels due to a disequilibrium between pro- and anticoagulant factors (5). Recently, a decreased protein C activation and hypercoagulability have been demonstrated in six patients with ALF (6). Although spontaneous bleeding is not directly related to commonly measured coagulation factors, the use of frozen plasma is mandatory before invasive procedures (i.e. intracranial pressure monitoring) as otherwise bleeding can occur (7).

Inflammation and endothelial damage could be additional factors impairing coagulation in these patients. Inflammation accompanied by a sepsis-like syndrome is common in ALF patients, particularly following paracetamol overdose (8). Natural anticoagulants [glycosaminoglycans (GAGs)] normally linked to the endothelium could be released by endothelial damage and could contribute to the coagulopathy as shown in patients with chronic liver disease (9), but this has never been evaluated in patients with ALF.

Thromboelastography (TEG™) is a rapid method to assess the entire coagulation process using whole blood. The addition of heparinase I, which cleaves heparin-like compounds, can reveal the presence of a heparin-like effect (HLE) due to an increased amount of endogenous anticoagulants (GAGs) (10). A HLE has been demonstrated in patients with liver disease and bacterial infections, correlating with an anti-Xa activity in some (9, 11).

A new development of TEG™ technology has allowed the production of a vcurve™ from the TEG™ trace (12). Previous work using thrombin–antithrombin measurements had postulated that thrombin generation is normal in cirrhosis and constant during orthotopic liver transplantation (OLT), although it may increase after reperfusion of the graft (13). The data produced by the vcurve™ is based on a mathematical model derived from the TEG™ data. The measurements produced from the vcurve™ (Haemoscope Corp, Skokie, IL, USA) software give an indication of the speed of the thrombin burst [time to maximal generation (TMG)] and its intensity [maximal thrombin generation (MTG), the peak of the thrombin generation curve] as well as the total thrombin generated (TTG, the area under the curve) and can be use as a surrogate marker of thrombin generation. These describe how the characteristics of thrombin generation differ when a patient is hypocoagulable, resulting in a longer TMG and a smaller MTG (12). This methodology has been evaluated against the standard thrombin–antithrombin enzyme-linked immunosorbent assay method of measuring thrombin generation and was found to have a high correlation (*r*=0.94) (14). Thus, the thrombin generation curve (vcurve) has potential in investigating the coagulation abnormalities observed in ALF.

The aim of our study was to evaluate the contribution of endogenous heparinoids evaluated by heparinase I-modified TEG™ in the coagulopathy of patients with ALF undergoing LT. This liver failure group was compared with a group of patients with cirrhosis undergoing LT and a control group of healthy volunteers.

## Methods

Thirty subjects were enrolled in this study; 10 patients with ALF and 10 patients with liver cirrhosis, all 20 undergoing LT. Ten healthy volunteers with no known haemostatic disorders and not taking medication having an influence on coagulation within the preceding 30 days were used as the control group.

History of recent bleeding, documented bacterial infections, presence of hepatocellular carcinoma and cholestatic aetiology of liver disease were exclusion criteria for the cirrhotic group, the latter two being known as procoagulant conditions. Patients with ALF and with cirrhosis who received blood products in the previous week were excluded from the study. All lines and flush bags during transplant were nonheparinized. None of the patients received protamine.

All the patients had given informed consent for collection of 10 ml of whole noncitrated blood into a noncitrated tube (Sarstedt Monovette® tubes, Sarstedt, Leicester, UK) with a 21 G needle with a light tourniquet, avoiding stasis and platelet activation. In ALF and cirrhotic patients, 10 ml of blood was collected from a dedicated nonheparinized arterial cannula at the stages detailed below. Samples were collected into a plain syringe and were not activated by celite/kaolin.

Three hundred and sixty microlitres of whole blood was used for TEG™ analysis and the remaining blood was centrifuged at 3000 *g* for 12 min at 4 °C. The plasma was then removed and centrifuged at 3000*g* for a further 12 min at 4 °C to obtain platelet-poor plasma. Samples were aliquoted into Sarstedt cryotubes (Sarstedt, Leicester, UK) and stored at −70°.

### Thromboelastography

Soon after the drawing of blood, 360 μl of whole blood was reverse pipetted into a plain and a heparinase-coated TEG™ cup. Samples were run simultaneously and immediately on blood collection as per manufacturer's recommendation. TEG™ was performed using a computerized thromboelastograph (Haemoscope Corp, Skokie, IL, USA).

The blood was mixed with the cuvette by lowering the pin three times and covered with mineral oil to prevent evaporation during analysis. The TEG™ was allowed to run till the maximum amplitude (MA) was reached.

Paired TEG™ traces (with and without heparinase) were examined at five stages before and during LT: 1, at baseline; 2, 1 h into the dissection; 3, half an hour into the anhepatic period; 4, half an hour after reperfusion; 5, at the end of the operation.

We evaluated the differences between the native TEG™ parameters and the same parameters obtained after the addition of heparinase I. The TEG™ parameters (*r*=reaction time, *k*=clotting time, α=alpha angle, MA=maximum amplitude) were generated and recorded automatically by a computer: *r* is the time from sample until the TEG™ trace amplitude reaches 2 mm, which represents the rate of initial fibrin formation and is functionally related to plasma clotting factors and circulating inhibitor activity; *k* is measured from *r* to the point where the amplitude of the tracing reaches 20 mm – it is the time taken to reach a standard clot firmness and is affected by the activity of the intrinsic clotting factors, fibrinogen and platelets; α is the angle formed by the slope of the TEG™ tracing from the *r* to the *k* value, representing the rate of clot growth and describing the polymerization of the structural elements involved in clotting; MA is the maximum variation on TEG™ parameters within the same group.

Definitions of HLE vary in the literature and, based on the percentage of correction of the TEG™ trace by addition of heparinase, we calculated the percentage correction of the *r*+*k* times on TEG™ with the addition of heparinase as shown: 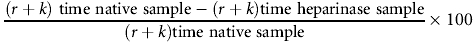


Heparin-like effect was defined as a correction of *r*+*k* times on TEG™ of more than 50% by the addition of heparinase. Those with greater than an 80% correction were categorized as demonstrating severe HLE.

The TEG™ traces and appropriate software (vcurve™) were used to derive the TTG at each stage according to the paper by Sorensen *et al.* (15). The TTG data were examined for differences in the native traces of those with and without HLE.

### Standard coagulation tests

The PT (rabbit HS-Plus-Fib; Instrumentation Laboratory, Warrington, UK), activated tissue thromboplastin time (APTT) (lyophilized APTT reagent; Instrumentation Laboratory) and thrombin time (bovine thrombin; Diagnostic Reagents, Thame, UK) were performed on stored plasma aliquots for each concentration of GAG using standard techniques with an ACL FUTURA coagulometer (Instrumentation Laboratory).

### Statistical analysis

The two-tailed nonparametric Wilcoxon matched-pairs test was used to compare the basal and heparinase I TEG™ at every stage during OLT. Differences of standard coagulation and TEG™ parameters between the three groups of patients were evaluated by the Kruskal–Wallis method and between the two groups by the Mann–Whitney *U* test; *P*<0.05 was considered statistically significant. For all calculations, spss for Windows version 10 (SPSS Inc., Chicago, Illinois, USA) was used.

## Results

### Patients

Ten patients with ALF, 10 patients with liver cirrhosis undergoing LT and 10 healthy volunteers as control group were enrolled. Characteristics of patients and controls are shown in Table 1.

**Table 1:** Characteristics of patients and of the control group

Groups	Acute liver failure	Cirrhosis	Controls
Number of subjects	10	10	10
Age (years ± SD)	42.3 ± 4	53.6 ± 6	38.5 ± 6.5
Sex (M/F)	4/6	7/3	8/2
Aetiology Alcohol/viral/cryptogenic/paracetamol	0/2/3/5	6/3/1/0	–
Acute/subacute form	**7/**3	–	–
Child–Pugh A/B/C	–	0/7/3	–
MELD 〈15/〉15	–	4/6	–

F, female; M, male; MELD, model for end-stage liver disease; SD, standard deviation.

### Standard coagulation and thromboelastographical parameters

Before skin incision, patients with ALF showed worse standard coagulation parameters compared with cirrhotic patients and with the control group.

Thromboelastography showed that patients with ALF had significant prolongation of *r* and *k* times, lower α angle and reduced MA at TEG™ compared with both the cirrhotic and the control groups. Details on standard coagulation tests and TEG™ parameters and significant differences are shown in Table 2.

**Table 2:** Standard coagulation parameters and thromboelastographical parameters and total thrombin generated derived using thromboelastography

	Acute liver failure	Cirrhosis	Controls	*P*
PT (s)	32.6 ± 18.1	19.7 ± 4.5	14.4 ± 0.2	0.029
INR	2.8 ± 1.4	1.6 ± 0.4	1 ± 0.2	0.009
PTT (s)	53.6 ± 15.2	42.7 ± 9.5	34.6 ± 1.4	0.040
PLT (10^9^/L)	114.8 ± 86.2	90 ± 11.3	234 ± 30	0.347
*r* (s)	49.9 ± 31.08	21 ± 5.5	18.7 ± 3.4	0.021
*k* (s)	22.8 ± 9.3	14.4 ± 9.3	27.4 ± 5.5	0.034
Angle (°)	7.5 ± 7.2	22.8 ± 9.3	27.5 ± 5.5	0.001
MA (mm)	38.9 ± 6.4	44 ± 9.1	47 ± 7.2	0.461
TTG (mm × 100)	5333 ± 976	5274 ± 1034	5574 ± 754	0.543

INR, international normalized ratio; *k*, clotting time; MA, maximum amplitude; PLT, platelets; PT, prothrombin time; PTT, partial thromboplastin; *r*, reaction time; TTG: total thrombin generated

Despite the different thromboelastographical parameters, analysis of the vcurve as surrogate of TTG revealed similar values among the three groups (Table 2).

### Reversal of clotting inhibition by heparinase I

**Fig. 1:**
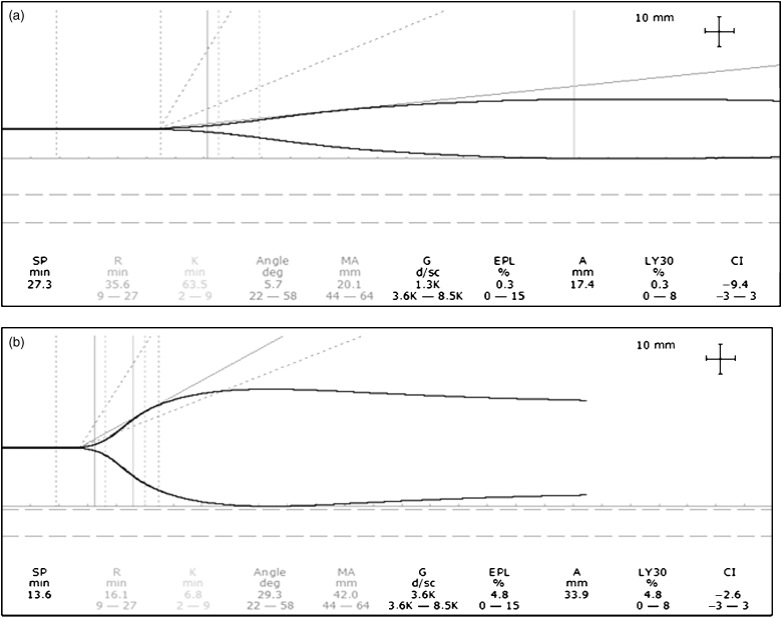
Native thromboelastography (TEG™) (a) and heparinase I TEG™ (b) in samples collected from a patient with acute liver failure at baseline before liver transplantation. Significant heparin-like effect is shown by the very slow rate of coagulation. Treatment of the sample with heparinase I significantly increased the rate of coagulation, indicating the presence of heparin-like substances.

Heparinase I-modified TEG™ demonstrated a HLE, by definition a correction of *r*+*k* times on TEG™ of more than 50% in one out of 10 patients with cirrhosis and nine out of 10 patients with ALF (*P*=0.0016; Fisher's exact test). Four of the nine patients with ALF and HLE had severe HLE (reduction of *r*+*k* times by addition of heparinase I>80%; Fig. 1a and b).

The differences (correction of *r*+*k* times) in heparinase I-modified TEG™ between cirrhotic and ALF groups were statistically significant before incision (stage 1) (*P*=0.01) and also during dissection (phase 2) (*P*=0.03). Patients with cirrhosis had no significant correction of TEG™ parameters after addition of heparinase I in all the stages before reperfusion, showing no HLE before transplantation. As expected, after reperfusion, HLE was severe in both cirrhotic and ALF patients (correction of TEG™ trace by more than 80% after addition of heparinase I).

**Fig. 2:**
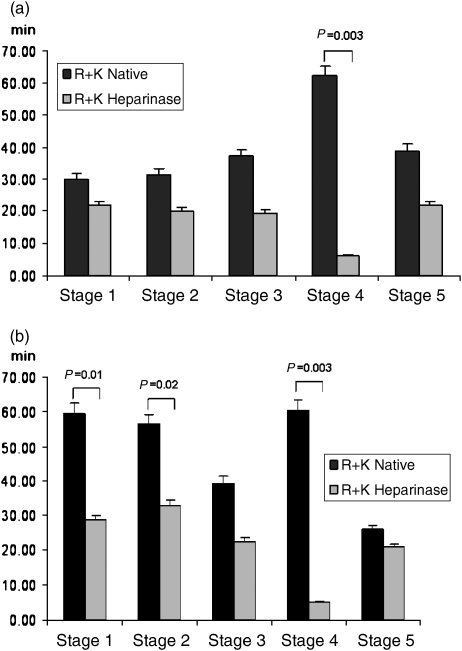
*r*+*k* times in native and heparinise I thromboelastography in patients with cirrhosis (a) and acute liver failure (b) at different stages during liver transplantation. Stage 1, baseline; stage 2, dissection; stage 3, anhepatic; stage 4, reperfusion; stage 5, end of operation.

A graphical representation of *r*+*k* times at baseline and during different stages during LT in native and heparinase I-modified TEG™ in ALF and cirrhotic patients is shown in Figure 2a and b.

As previously reported (20), there was no difference between TEG™ and heparinase I-modified TEG™ parameters in healthy controls (*r* 18.7±2.4 seconds vs 20.6±5.4 seconds; *k* 7.5s±3.6 seconds vs 6.8±2.3 seconds; α angle 27.4±4.8° vs 29.8±5.5°: MA 47.6±7.9 vs 49.6±8.6 mm).

**Fig. 3:**
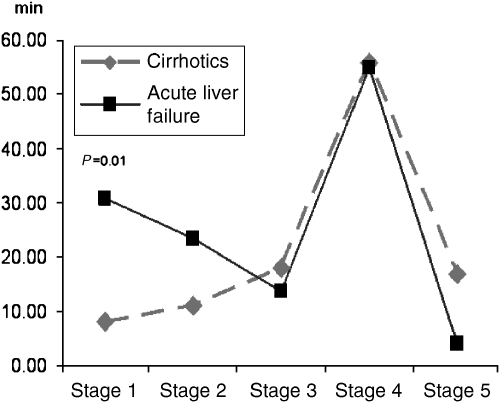
Comparative representation of heparin-like effect shown by the difference between native and heparinase I thromboelastography *r*+*k* times at different stages during orthotopic liver transplantation between cirrhotic and acute liver failure patients. Stage 1, baseline; stage 2, dissection; stage 3, anhepatic; stage 4, reperfusion; stage 5, end of operation.

When the magnitude of the effect of heparinase I was compared between ALF and cirrhotic patients, ALF patients had a greater difference between the *r*+*k* times native and after the heparinase I addition, before incision and during the dissection phases (Fig. 3).

## Discussion

We report for the first time the contribution of endogenous heparinoids to the abnormalities in coagulation of patients with ALF who require LT. ALF is characterized by a reduced synthesis of all coagulation factors, both pro- and anticoagulant, but the von Willebrand factor and factor VIII are released by the endothelium (16). In particular, factor VIII is increased because it is released by endothelial cells during the underlying inflammation process.

Inflammation and increased levels of interleukin-6 and tumour necrosis factor-α seem to have an important role leading to increased synthesis of tissue factor and promoting thrombin formation (17).

Increased release of cytokines can lead to endothelial injury together with the sepsis-like syndrome [systemic inflammatory response syndrome (SIRS)], which is well documented in ALF. This could lead to the release of endogenous heparinoids, which are synthesized by endothelial cells and are naturally bound to the endothelium of the vessels, preventing spontaneous thrombosis (18). Moreover, during sepsis or SIRS, mast cells can release heparin-like substances (19). For the first time, we reported a HLE in 90% of the patients with ALF, demonstrating that endogenous GAGs are responsible for a major part of anticoagulation. In about 50% of these, addition of heparinase I was able to correct the trace by more than 80%, as shown in Figure 2. Which heparinoid (GAGs) is wholly or in a major part responsible for this effect cannot be derived from our data. We have already shown that heparinase I is able to detect not only the effect of heparan sulphate, which is the major natural anticoagulant lining the endothelium, but also of dermatan sulphate (20).

Glycosaminoglycans (heparinoids) are constituents of the vessel wall and can be bound by the endothelium. Endothelial cells synthesize heparan sulphate and are also able to bind other heparin-like substances, such as dermatan and chondroitin sulphate (21). All these GAGs have anticoagulant properties and help to maintain coagulation haemostasis at the endothelial surface. Heparan sulphate is the most important GAG, but dermatan sulphate has recently been recognized to have important anticoagulation properties. Dermatan sulphate inhibits thrombin generation by forming an inactive ternary complex with heparan cofactor II (20). It has also been shown to be as effective as heparin in preventing post-operative thrombosis and it can be used when heparin-induced thrombocytopenia occurs.

During sepsis, sepsis-like syndrome or infection, the liver is exposed to a neutrophil-mediated injury, involving both hepatocytes and endothelial cells, which could release heparin-like substances (heparan sulphate) into the systemic circulation (22), analogous to what happens during suramin treatment in patients with metastatic liver disease (23). It is well known that the liver contains abundant parenchymal deposits of GAGs, heparan sulphate being predominant (24).

Additionally, and particularly in patients with ALF, the ability to eliminate circulating GAGs is likely to be greatly reduced due to the important reduction of liver function. McKee *et al.* (25) have shown a reduced hepatic clearance of heparin in patients with liver cirrhosis and increased serum levels of heparin sulphate after variceal bleeding. Although sepsis like syndrome could be an additional factor responsible for the release of GAGs from the endothelium, the patients in the ALF group did not show significantly different haemodynamic parameters from cirrhotic patients, although there was a trend towards a more ‘septic’ picture, with a lower systemic vascular resistance and a higher cardiac index (data not shown).

In our study, patients with ALF had worse standard coagulation parameters as expected, compared with both the cirrhotic and the control group, reflecting poor synthetic hepatic synthesis of coagulation factors (more than haemostatic balance), which was reflected in the abnormal thromboelastographical parameters. However, despite a slower abnormal velocity of formation of the clot, vcurve (calculated by TEG™) was not different either from the controls or from the cirrhotics. This may be due to the fact that platelets are essential in thrombin formation and MA (which correlates with platelet function) was not found to be significantly different between the groups (26). Sorensen *et al.* (15) used various amount of tissue factor (in contrast to our study in which none was used) to activate the clotting process in the TEG™ cups, resulting in different times to reach the maximum amount of calculated generated thrombin, but it did not result in a significantly different generation of total thrombin. A validation of TEG™ as a whole blood test to calculate thrombin generation is needed and the significance of normal thrombin generation in liver disease is still to be evaluated clinically, given the bleeding tendency with invasive procedures.

As expected and already reported, during LT, the coagulation profiles of both ALF and cirrhotic patients show greater HLE after reperfusion, which subsides by the end of the operation (27). Estimated thrombin generation after reperfusion was reduced compared with baseline and was similar between cirrhotics and ALF patients (2430 × 100 mm vs 2198 × 100 mm respectively).

It is well known that during invasive procedures, patients with ALF have an increased risk of bleeding and correction of coagulation is indicated (7). Commonly, fresh frozen plasma is used to correct coagulation in patients with ALF undergoing invasive procedures and, recently, recombinant thrombin factor VII has been tried to accelerate the coagulation process (28).

The potential therapeutic implications of our finding of a significant HLE is not clear at present. The use of protamine in cases where HLE is present is controversial. Pivalizza *et al.* (29) and Bayly and Thick (30) have used protamine *in vivo*, demonstrating the reversal of HLE in patients with liver cirrhosis undergoing LT. The neutralization of the heparin effect could decrease the peri-operative blood loss, but this is controversial. Although the neutralization of the HLE could decrease the peri-operative blood loss, hypercoagulation can be induced, increasing the risk of vessel thrombosis and the consequent graft loss (31). The use of specific drugs (i.e. Neutralase), which selectively cleave heparinoids, has not been reported in LT.

We conclude that the presence of HLE contributes significantly to the coagulopathy of patients with ALF, and this is not seen in patients with stable liver cirrhosis. Both groups exhibit normal thrombin generation. The clinical significance of the HLE in relation to bleeding tendency is not known and needs to be formally explored. Further work is needed regarding thrombin generation, which may not be the correct parameter to understand coagulopathy in patients with liver cirrhosis and ALF.
